# Should cannabis be legalised?

**DOI:** 10.1192/j.eurpsy.2025.254

**Published:** 2025-08-26

**Authors:** R. Keet

**Affiliations:** FIT-academy, GGZ Noord-Holland-Noord, Heerhugowaard, Netherlands

## Abstract

**Abstract:**

Legalizing cannabis can yield significant benefits for public mental health by fostering harm reduction, promoting medical access, and mitigating societal stigma. While concerns about misuse exist, a well-regulated cannabis policy can outweigh these risks and provide a balanced approach to mental health promotion. 1. Harm Reduction through Regulation Prohibition often drives cannabis use into unregulated markets, where the lack of quality control increases risks of contamination with harmful substances. Legalization allows governments to regulate cannabis production, ensuring product safety and controlled potency. This can reduce incidents of adverse reactions, particularly those exacerbated by high-potency strains or toxic additives. A regulated market also discourages illicit activity, reducing exposure to dangerous drugs often sold alongside cannabis in black markets. 2. Promoting Medical Access and Mental Health Treatment Legalization enhances access to cannabis for therapeutic purposes, particularly for mental health conditions such as chronic pain and opens the door to explore the potential benefor for patents with anxiety and post-traumatic stress disorder (PTSD), and. Studies indicate that cannabinoids can alleviate symptoms of stress and anxiety when used responsibly and under medical supervision. By integrating cannabis into healthcare systems, individuals struggling with mental health disorders can benefit from a natural and potentially less addictive alternative to pharmaceuticals like opioids or benzodiazepines, which carry significant risks of dependency. 3. Addressing Stigma and Encouraging Open Dialogue Legalization reduces societal stigma associated with cannabis use, enabling more individuals to openly discuss their experiences and seek help for misuse if needed. Public health campaigns can then focus on education about responsible use, mental health implications, and support systems. Decriminalizing cannabis also reduces the disproportionate criminalization of marginalized groups, fostering a more inclusive and equitable approach to public health. 4. Potential to Reduce Alcohol and Opioid Use Research has suggested that legal cannabis availability is associated with reductions in alcohol and opioid consumption, substances that are more harmful to both physical and mental health. By providing a less harmful alternative for relaxation or pain management, cannabis legalization could mitigate the societal burden of these substances, including addiction and overdose crises. Conclusion While cannabis legalization requires careful regulation to mitigate risks like overuse or dependence, its potential benefits for public mental health are substantial. By reducing harm, enhancing medical access, and promoting a more informed and equitable societal approach, legalization represents a forward-thinking public health policy that prioritizes well-being over punitive enforcement.

**Disclosure of Interest:**

None Declared

